# The contribution of contraception, marriage and postpartum insusceptibility to fertility levels in Uganda: an application of the aggregate fertility model

**DOI:** 10.1186/s40738-015-0009-y

**Published:** 2015-10-17

**Authors:** Gideon Rutaremwa, Johnstone Galande, Hellen Laetitia Nviiri, Edith Akiror, Tapiwa Jhamba

**Affiliations:** 1 Social Development Policy Division, United Nations Economic Commission for Africa (ECA), PO Box 3001, Addis Ababa, Ethiopia; 2 Uganda Bureau of Statistics, Ministry of Finance and Planning, Kampala, Uganda; 3 United Nations Population Fund (UNFPA), Uganda Country Office, Kampala, Uganda

**Keywords:** Contraception, Marriage, Postpartum infecundability fertility, Aggregate fertility model, Uganda

## Abstract

**Background:**

While recent studies have indicated that fertility has remained high in Uganda, no systematic attempt has been made to identify the factors responsible for this persistent trend and to quantify these factors. This paper uses the Uganda Demographic and Health Surveys (UDHS) of 2006 and 2011, to examine the contribution contraceptive use, marriage and postpartum infecundability on one hand and Total Fertility Rate (TFR) on the other.

We constructed a database using the Woman’s Questionnaire from the UDHS 2006 and 2011. We then apply Bongaarts aggregate fertility model procedures to derive estimates of total fertility rate for the different socioeconomic groups.

**Results:**

The findings indicate that a woman’s contraceptive behavior; marriage status and postpartum infecundability (also referred to as postpartum insusceptibility due to postpartum amenorrhea, which is intended to measure the effects on fertility breastfeeding), are important predictors of fertility outcomes. The results also show that higher education levels and urban residence are consistently associated with lower fertility rates and are positively associated with contraceptive use. Other key predictors of fertility include: wealth status, and region of residence.

**Conclusion:**

The country needs to scale-up target interventions that are aimed at uplifting the education status of women and improving their economic wellbeing, because such interventions have a positive impact on fertility reduction and on improving maternal and reproductive health outcomes.

## Background

Reduction of total fertility rates (TFR) is a key determinant of overall reduction in population growth and transition from high to low fertility. These in turn may have important consequences for economic growth, poverty reduction, and improved health and nutrition outcomes [1]. Uganda currently has one of the highest fertility rates in the world, although there are marked differences between rural and urban fertility rates. In sub-Saharan Africa, the prevalence of contraceptive practice is low, and fertility levels are exceptionally high for recorded levels of contraceptive practice, even where levels of contraceptive practice are comparable to other regions. In most resource poor countries, particularly sub-Saharan Africa, modern contraceptive use and prevalence are unusually low and fertility is very high resulting in rapid population growth and high maternal mortality and morbidity [2, 3]. In the latter study rural–urban residence gap in the use of modern contraceptive methods had almost disappeared in 2008, while education and income related inequalities remained.

Socio-demographic characteristics of mothers like poor educational status, absence of income, rural place of birth, early marriage, and other variables like history of child death, negative husbands’ attitude towards contraceptive use, poor educational status of husbands, need for additional children, were found to have significant association with high fertility in Ethiopia [4–8].

Other than contraceptive use, the fertility differences between population groups can be explained by the variations in other proximate determinants; notably exposure to the risk of pregnancy, and abstinence after delivery [9]. Unmet need for modern contraceptives and unintended pregnancy levels remain significant [10]. In Uganda unmet need for family planning currently stands at 34.3 % [11]. An analysis of the proximate determinants shows that the difference was primarily due to greater contraceptive use in Kenya; though in Uganda there was also a reduction in pathological sterility. The Demographic and Health Surveys showed that women in Kenya wanted fewer children than those in Uganda, but that in Uganda there was also a greater unmet need for contraception [12].

There is now widespread agreement on the importance of men’s role in reproductive decision-making. Several studies have argued that fertility preferences and their translation into behavior differ between polygamous and monogamous unions [13]. Studies investigating the dominance of men’s preferences over women’s preferences, in cases of couple disagreement, found mixed evidence of the effect of polygamy. In a related study, the contribution of adolescent fertility to total fertility and mortality is said to remain quite high, while delayed marriage is occurring concomitantly with postponement of sexual debut may act to reduce the total achieved fertility [14].

Young women aspire to have an ideal ordering of events that places finishing education before getting married and having children, but this is often not easily attained [6, 15]. There are important differences in the ways young women and their families respond to union formation and childbearing that often occurs outside of a recognized union [15]. In Botswana, marriage was the least important proximate determinant of fertility, probably due to the high prevalence of premarital childbearing [16]. In another study using the Ghana Demographic and Health survey data, authors provide evidence that couples adjust their coital frequency in accordance with their fertility preferences, behaviour that would influence fertility rates but would not be captured by conventional measures of the proximate determinants of fertility [17].

While recent studies have indicated that fertility has remained high in Uganda, no systematic attempt has been made to identify the factors responsible for this persistent trend and to quantify these factors. Therefore the current study was based on the Bongaarts aggregate fertility model using data from two most recent DHS surveys of the Uganda: 2006 and 2011. This study explored the key drivers of fertility differences, including systematic analysis of the key proximate determinants, namely: contraception, marriage and postpartum insusceptibility.

## Methods

### Data sources

As noted earlier the data were extracted from the Uganda Demographic and Health Surveys conducted in 2006 and 2011. Approval for UDHS data utilized for this study was obtained from the data originator, ICF Macro International U.S.A before the data was extracted from their web platform. At the point of data collection by the data originators, an informed consent was sought from all the study participants after detailed description of all the issues related to the study were passed across to the respondents. Eligible respondents who did not want to participate in the study were excluded from the survey. Each consenting participants was made to sign appropriate agreement form before the commencement of the interview. These surveys employed nationally representative samples, which were based on a two-stage stratified sample of households.

These types of surveys generally provide information on basic national indicators of social development. The present study utilised these data in order to fit the aggregate fertility model, thereby assessing the contribution of contraception, marriage and postpartum infecundability to fertility in Uganda. The fertility estimates were also disaggregated by a number of selected variables, namely: education, religion, residence, region of residence, and wealth index.

### Sampling

A total of 8674 and 8531 women of ages 15–49 from the 2006 and 2011 Uganda Demographic and Health Surveys respectively were selected for the current study. In 2011 the household response rate was 97.4 % while the eligible women response rate was 93.8 %. Furthermore in 2006 the household response rate was 97.5 % while the eligible women response rate was 94.7 %. These samples are considered adequate to enable analyses and comparisons that would be useful in the identification of socio-economic and regional foci that could guide fertility and population policy interventions in Uganda.

### Analysis method

#### The Bongaarts model

The Bongaarts model is adopted to quantify the contribution of the proximate determinants to fertility. Bongaarts’ original model included four proximate determinants: marriage, postpartum infecundability, abortion and contraception. In a later paper, Bongaarts added a fifth determinant, pathological sterility [18, 19]. The basic model is:

1 $$ \mathrm{T}\mathrm{F}\mathrm{R} = {\mathrm{C}}_{\mathrm{m}}*\ {\mathrm{C}}_{\mathrm{i}}*\ {\mathrm{C}}_{\mathrm{a}}*\ {\mathrm{C}}_{\mathrm{c}}*\ \mathrm{T}\mathrm{F} $$

where C_m_ is the index of proportion married, C_i_ is the index of lactational infecundability, C_a_ is the index of abortion, C_c_ is the index of contraception and TF is total fecundity. The indices can only take values between 0 and 1. When there is no fertility-inhibiting effect of a given intermediate fertility variable, the corresponding index equals 1. If the fertility inhibition is complete, the index equals 0. These indices can be estimated from measures of the proximate variables. Although this aggregate version of the model is the most widely used, there is also an age-specific version that calculates the effects separately for each five year age group from 15–19 to 45–49 [20].

### The index of marriage (Cm)

The age-specific proportions of determine the index of marriage currently married among females. The index Cm is not simply equal to the proportion of all women of reproductive age that is married because the fertility impact of marriage also depends on the age distribution of married women. Married women in the central childbearing years contribute more to the TFR than the youngest or oldest women because the age-specific marital fertility rates reach their maximum in the central childbearing ages. The index is equal to the ratio of the total fertility rate to the total marital fertility rate. The index of marriage equals one when all women of reproductive age are in a union and zero when no women are in a union. Marriage in this context refers to both formal marriage and consensual unions. Implicit in the use of the index is the assumption that only women in union are exposed to the risk of childbirth. This assumption does not hold reasonably well in Uganda where childbearing outside of marriage do exist.

2 $$ \begin{array}{l}{\mathrm{C}}_{\mathrm{m}}=\Sigma \left(\ \mathrm{m}\mathrm{a}\right)\ \left(\ \mathrm{g}\mathrm{a}\right)\\ {}\kern2.5em \Sigma \left(\ \mathrm{g}\mathrm{a}\right)\end{array} $$

Where: m(a) = age-specific proportions of women currently married (it is got by dividing the number of married women of a particular age group by the number of women in the same age group); g(a) = age-specific marital fertility rate (is got by dividing the births of a particular age group (from married women) by the number of married women in the same age group).

### The index of contraception (C_c_)

The index of contraception (C_c_) varies inversely with prevalence and use-effectiveness of contraception practiced by couples in the reproductive age groups. It incorporates both prevalence of contraceptive use and estimated effectiveness of the mix of methods used. It equals one if no form of contraception is used and zero if all fecund women use modern methods (modern methods included pill, IUD, injection, diaphragm, condom, sterilization, implant, or foam/jelly) that have a higher effectiveness [21].

3 $$ {\mathrm{C}}_{\mathrm{c}} = 1\ \hbox{-}\ 1.08\mathrm{u}\mathrm{e} $$

Where: u = the average proportion of married women currently using contraception; e = the average use contraceptive effectiveness–which measures how well a contraceptive method works in typical use; and 1.08 is the sterility correction factor (represents an adjustment for the fact that women do not use contraception if they know that they are sterile).

### The index of postpartum infecundability (Ci)

The index of postpartum infecundability is a measure of the inhibiting effect of breastfeeding or abstinence on fertility in the population [22]. The index of postpartum infecundability in the model is estimated using the effect of breastfeeding (lactation amenorrhea) or postpartum abstinence. The index of infecundability, Ci, is calculated using the mean number of months of postpartum infecundability. It equals one in the absence of breastfeeding and postpartum abstinence and zero when infecundability is permanent. If no breastfeeding and postpartum abstinence are practiced, the birth interval averages about 20 months, the sum of 1.5 months of minimum postpartum an ovulation, 7.5 months of waiting time to conception, 2 months of time added by spontaneous intrauterine mortality, and 9 months for a full term pregnancy. In the presence of breastfeeding and postpartum abstinence the average birth interval equals, approximately 18.5 months (7.5 + 9 + 2) plus the duration of postpartum infecundability. Then index Ci is estimated as;

4 $$ \mathrm{Ci} = 20\ /\ \left(18.5+\mathrm{i}\right) $$

Where: i = the mean duration of postpartum infecundability measured in months. According to Bongaarts, without lactation, a typical average birth interval is estimated at 20 months, and with lactation it equals the average total duration of the infecund period plus 18.5 months.

### The index of abortion (Ca)

Abortion is illegal in Uganda. The total abortion rate can be used to assess the relationship between induced abortions and fertility. This rate is equivalent to the TFR but includes only induced abortions (rather than births) in the numerator. Because of lack of reliable data for induced abortion, the index of abortion was estimated to 1. The difficulty of getting such data was reported by Bongaarts [23], and according to the Bongaarts model, fertility differences among populations and trends in fertility over time can always be traced to variations in one or more of the proximate fertility variables. Therefore, the index of abortion was not computed. The 2006 and 2011 had questions on abortion but they contained still births and miscarriages combined [24], and therefore are not appropriate for use in these analyses.

The index of abortion is estimated using the formula below;

5 $$ \begin{array}{l}\mathrm{C}\mathrm{a} = \mathrm{T}\mathrm{F}\mathrm{R}/\left(\mathrm{T}\mathrm{F}\mathrm{R} + \mathrm{b}\ \mathrm{T}\mathrm{A}\right)\\ {}\kern1.5em  = \mathrm{T}\mathrm{F}\mathrm{R}/\mathrm{T}\mathrm{F}\mathrm{R} + 0.4*\left(1+\mathrm{u}\right)*\mathrm{T}\mathrm{A}\end{array} $$

Where u = Prevalence contraceptive use; b = Average number of births averted per induced abortion and b = 0.4 (1 + u); b = 0.4 when u = 0 and b = 0.8 when u = 1.0.TA = Total abortion (Average number of induced abortions per woman at the end of the reproductive period if induced abortion rates remains at prevailing levels throughout the reproductive period). Therefore, C a = 1.0 if the TA is 0. Therefore the Total Abortion rate in this study is assumed to be 1.0.

When all indices equal one, fertility is at its biological maximum. Based on studies of historical populations with the highest recorded fertility, Bongaarts recommends using 15.3 as the maximum number of births per woman; this is referred to as the total fecundity rate [23]. This value is the theoretical number of births that a woman would have if she were continuously married from age 15 to 44, did not use contraceptives, did not breastfeed and did not abort any pregnancies. Multiplying all of the indices together by the total fecundity rate of 15.3 produces the predicted TFR for the population. The predicted TFR will typically differ from the observed TFR because of the underreporting of births; misreporting of behaviors measured by the indices; or omission of proximate factors that help determine fertility levels in the population under study.

## Results

### Descriptive findings

Table 1 shows the individual characteristics of women respondents. The findings suggest that about 80 % of the sample women in 2006 and in 2011 were from rural areas. In terms of regional distribution there were wide variations in the contribution to the overall study population ranging from as low as 9.7 % in Kampala to 26.7 % in Western Uganda in 2011. As of 2006 UDHS, the region with the smallest sample was again Kampala (8.5 %), while the one with the highest proportion was again Western region (27.6 %). Education level attainment is another factor that is known to influence the fertility of the woman [3, 4, 6, 8]. In 2006 19.3 % of the respondents had no education, this percentage reduced to only 12.9 % in 2011. The latter appears to be driven by the improvements in access to secondary education. Therefore, women who had at least a secondary education increased from 21 % in 2006 to nearly 28 % in 2011. The distribution of the study population by religious affiliation shows that about 85 % of the respondents were Christian in 2006 and about the same proportion was in this category in 2011.

**Table 1:** Weighted percentage distribution of respondents by selected characteristics

Variable		2011 UDHS		2006 UDHS	
	Category	Number	Percent	Number	Percent
Children born	1–3	4,918	56.7	4740	55.6
	4–6	2,114	24.4	2094	24.6
	7+	1,642	18.9	1697	19.9
Residence	Urban	1,717	19.8	1442	16.9
	Rural	6,957	80.2	7089	83.1
Region	Kampala	839	9.7	722	8.5
	Central	1,857	21.4	1,675	19.6
	Eastern	2,135	24.6	1,984	23.3
	North	1,524	17.6	1,793	21.0
	Western	2,319	26.7	2,357	27.6
Education level attainment	No education	1,120	12.9	1,650	19.3
	Primary	5,152	59.4	5,062	59.3
	Secondary	1,949	22.5	1,488	17.5
	Higher	454	5.2	331	3.9
Religion	Catholic	3,524	40.6	3614	42.4
	Protestant	3,754	43.3	3632	42.6
	Muslim	1,124	13.0	956	11.2
	Others	272	3.1	330	3.9
Marital Status	Never married	2,118	24.4	2,028	23.8
	Married	5,418	62.5	5,337	62.6
	Formerly married	1,134	13.1	1,167	13.7
Wealth Index	Poorest	1,519	17.5	1541	18.1
	Poorer	1,579	18.2	1636	19.2
	Middle	1,608	18.5	1615	18.9
	Richer	1,726	19.9	1621	19.0
	Richest	2,242	25.9	2118	24.8
Age group	15–19	2,048	23.6	1936	22.7
	20–24	1,629	18.8	1710	20.0
	25–29	1,569	18.1	1413	16.6
	30–34	1,086	12.5	1217	14.3
	35–39	1,026	11.8	940	11.0
	40–44	729	8.4	735	8.6
	45–49	587	6.8	580	6.8
Amenorrhea duration	0–4 months	1,675	19.3	1,477	17.3
	5–8 months	831	9.6	812	9.5
	9 + months	1,779	20.5	2,048	24.0
	All other	4,389	50.6	4,194	49.2
Type of Family Planning^a^	All other	6,880	79.3	7213	84.6
	Modern	1,794	20.7	1318	15.4
TOTAL (N)		8,674		8,531	

Note: ^a^The modern methods included pill, IUD, injection, diaphragm, condom (male or female), sterilization (male or female), implant, or foam/jelly), while all other methods include the rest such as rhythm, folk methods and traditional

The distribution of the study population by marital status shows that the proportion currently married was 62.6 % in 2006 and this stayed nearly the same in 2011, while those who were single at the time of the survey were only about 24 % in the two surveys. About one quarter of the women respondents in both 2006 and also in 2011 were in the richest wealth quintile, while about 42 % of the respondents were in the lowest two wealth quintiles in both years. Similarly those women in the middle wealth quintile held steadily at about 19 % in both 2006 and 2011. The distribution of the study population by age distribution clearly reflects a similar distribution for both surveys. About one fifth of the study population was aged 15–19 years and the proportions at each age group gradually declined reaching about 7 % in the age group 45–49 years.

Table 1 also shows that the distribution of the study population by some key proximate determinants of fertility About 21 % of women in 2011 were using modern contraceptives, increasing from 15 % in 2006. Concerning abstinence, about 4 % of women had durations of 0–4 months in both 2006 and in 2011. The category all other represents all other women who had not had a child in the 5 years preceding the survey and these comprised nearly 45 % in 2006 and 49 % in 2011. With regard to postpartum amenorrhea, 17 % had duration of 0–4 months in 2006 and this increased to 19 % in 2011. The category “all other” represented women who had not produced a child in the 5 years preceding the survey and this category comprised about half of the study population.

### Effect of contraception, marriage and postpartum infecundability on fertility

Tables 2 and 3 show the effects of various proximate factors on fertility levels in Uganda for 2006 and 2011, respectively.

**Table 2:** Estimated indices of proximate determinants of fertility for selected variable (2006)

Variable	Category	C_m_	C_c_	C_i_	C_a_	TFR
Religion	Catholic	0.756688	0.81124	0.72359	1.0	6.8
	Protestant	0.688570	0.76740	0.74156	1.0	6.0
	Moslem	0.723129	0.71102	0.75700	1.0	6.0
	Other	0.677555	0.78402	0.73206	1.0	5.9
Region	Kampala	0.532694	0.52907	0.81400	1.0	3.5
	Central	0.680401	0.66009	0.76805	1.0	5.3
	Eastern	0.776696	0.79588	0.74267	1.0	7.0
	Northern	0.773491	0.90037	0.69493	1.0	7.4
	Western	0.749218	0.77022	0.73475	1.0	6.5
Residence	Rural	0.761407	0.81240	0.72966	1.0	6.9
	Urban	0.562910	0.56882	0.79239	1.0	3.9
Wealth Index	Poorest	0.815166	0.92263	0.71200	1.0	8.2
	Poor	0.767808	0.85478	0.71023	1.0	7.1
	Middle	0.739220	0.81104	0.71968	1.0	6.6
	Richer	0.759015	0.73921	0.75614	1.0	6.5
	Richest	0.577128	0.53849	0.79618	1.0	3.8
Education	None	0.830933	0.89279	0.70547	1.0	8.0
	Primary	0.748226	0.78791	0.73233	1.0	6.6
	Secondary +	0.611473	0.56484	0.79904	1.0	4.2
TOTAL	UGANDA	0.722717	0.78179	0.73502	1.0	6.4

Note: *C*_*m*_ Index of marriage, *C*_*c*_ Index of contraception, *C*_*i*_ Index of postpartum infecundability, *C*_*a*_ Index of abortion, *TFR* Total fertility rate

**Table 3:** Estimated indices of proximate determinants of fertility for selected variable (2011)

Variable	Category	C_m_	C_c_	C_i_	C_a_	TFR
Religion	Catholic	0.739750	0.75710	0.75103	1.0	6.4
	Protestant	0.686338	0.68546	0.77042	1.0	5.5
	Moslem	0.765173	0.70558	0.77160	1.0	6.4
	Other	0.796222	0.65513	0.75729	1.0	6.0
Region	Kampala	0.568926	0.54316	0.83333	1.0	3.9
	Central	0.718897	0.64366	0.79491	1.0	5.6
	Eastern	0.758391	0.74057	0.77042	1.0	6.6
	Northern	0.723603	0.84672	0.72150	1.0	6.8
	Western	0.721989	0.68546	0.75358	1.0	5.7
Residence	Rural	0.780517	0.76701	0.74822	1.0	6.9
	Urban	0.592744	0.57047	0.81136	1.0	4.2
Wealth Index	Poorest	0.821068	0.89191	0.73233	1.0	8.2
	Poor	0.797797	0.78723	0.73665	1.0	7.1
	Middle	0.749112	0.71637	0.73719	1.0	6.1
	Richer	0.741234	0.66709	0.77131	1.0	5.8
	Richest	0.584853	0.54365	0.81934	1.0	4.0
Education	None	0.820628	0.87626	0.72595	1.0	8.0
	Primary	0.765763	0.72988	0.75047	1.0	6.4
	Secondary +	0.598991	0.56241	0.82203	1.0	4.2
TOTAL	UGANDA	0.724311	0.71870	0.76190	1.0	6.1

Note: *C*_*m*_ Index of marriage, *C*_*c*_ Index of contraception, *C*_*i*_ Index of postpartum infecundability, *C*_*a*_ Index of abortion, *TFR* Total fertility rate

#### Contraception (Cc)

Results presented in Tables 2 and 3 show that the overall the net inhibiting effect of contraception on fertility increased from 0.22 in 2006 to 0.28 in 2011. Overall the effect of contraception was greatest in 2011, but it was not the case in 2006, when the marriage effect was the greatest. The latter is shown by the value of Cc of 0.72 in 2006 and 0.78 in 2011, respectively. In 2011, the effect of contraceptives on fertility varied significantly and was particularly greater in Kampala region (Cc = 0.543). This effect was equally lower in Kampala region compared to other regions in 2006 (Cc = 0.529) followed by Central region with a Cc index of 0.660 in the same year. Northern region remained with the least contraceptive effect in both 2006 and 2011 and was followed by Eastern region for both years.

The rural/urban residential distribution shows that in 2006 and 2011, the contraceptive use index was better for urban areas relative to rural areas, with rural areas exhibiting some slight improvement compared to the urban areas at the two survey periods. The findings also suggest that contraceptive use was slightly higher among Protestants and Moslem religions in 2006 (Cc = 0.78 and 0.71), respectively. In 2011 there were some improvements in contraceptive use and some differences with Protestants and Other religions depicting better contraceptive use indicators (Cc = 0.68 and 0.66), respectively. In 2011, Moslems had a Cc of 0.71, which was the same as that of 2006.

Contraceptive index (Cc) was least among women in the richest wealth quintile for both 2006 and 2011 (Cc = 0.54), for both years respectively. Women in the poorest wealth quintile had the highest Cc index of 0.85 and 0.89 in 2006 and 2011, respectively, suggesting that contraceptive use effect on fertility was least among the poorest categories of the population compared to the richest individuals. Finally, the contribution of contraceptives to fertility inhibition was highest among women with a secondary and higher education compared to other education categories in both 2006 and 2011. The value of Cc held steadily at 0.56 at both survey periods, while slight improvements were observed among those with no education and those with a primary education during the same period.

#### Marriage index (Cm)

The index of marriage had the greatest inhibiting effect on fertility in Uganda in 2006 (Cm = 0.72). As is the case with the index of contraception, a higher value of Cm index is consistent low effective impact on fertility reduction. The value of Cm was the same in 2011 and had nearly same effect as Cc in the same year. The high contribution of the index of marriage to fertility reduction was especially substantial in Kampala region (Cm = 0.57), compared to other regions of the country. Eastern region depicted the highest index on marriage at 0.76. Similarly the index of marriage (Cm) was least among those residing in the rural areas (Cm = 0.78) compared to those in urban areas (Cm = 0.59).

Concerning the wealth index, the index of marriage was least among women in the richest wealth quintile (Cm = 0.58) in both 2006 and in 2011. The marriage index was highest among those in the poorest category at 0.78 and 0.82 in 2006 and 2011, respectively. Overall the findings in Tables 2 and 3 show an inverse relationship between the wealth quintile and the value of the marriage index. The last variable is educational level attainment, which also exhibits an inverse relationship between educational level of woman and value of marriage index. Women with a secondary and higher level of education had a lower marriage index of 0.61 in 2006 and 0.60 in 2011. Among those women with no education, the marriage index was 0.83 in 2006 and was 0.82 in 2011.

#### Postpartum infecundability (Ci)

The index of postpartum infecundability provided the least overall inhibiting effect on fertility compared to Cm and Cc in 2011 (0.76), but performed better in 2006 (Ci = 0.74). The results generally show that religious variations in the value of this index were minimal, ranging from 0.72 among Catholic’s to 0.76 among Moslems in 2006, and from 0.75 to 0.77 among the same groups, respectively in 2011.

Regional differences in the Ci suggest that Kampala, the capital city, had the highest value of the index at 0.81 and 0.83 in 2006 and 2011, respectively, while Northern region had the least value of the index at 0.79 and 0.72 at the two respective periods. The rural urban dimensions show that urban areas consistently had a high value of Ci compared to rural areas. In 2006 the Ci for urban areas of Uganda was 0.79 and was estimated at 0.81 in 2011.

The differences in the value of Ci were apparent for the various wealth index categories, but clearly, there was a direct relationship between wealth quintile and the value of Ci for both 2006 and 2011. This direct relationship was also observed for the education level attainment categories. Women with a secondary and higher education depicted the highest values of Ci (0.82 in 2011 and 0.80 in 2006) compared to those with no education (0.72 in 2011 and 0.71 in 2006).

#### Total fertility rate (TFR)

The aggregate fertility model findings presented in Tables 2 and 3 show that the overall TFR was 6.4 in 2006, declining to 6.1 in 2011. The findings also show variations in the TFR according to the selected variables. A few noticeable patterns are as follows: First, in 2006 Catholics depicted the highest TFR of 6.8 children compared to all other religious groups, whose TFR averaged about 6.0 children. In 2011 some slight differences from the 2006 rates were observed, again Catholics and Moslems had the highest TFR of 6.4, while Protestants had the least TFR of 5.5 children.

Regional differences in TFR were observed for both 2006 and 2011. Kampala district had the least TFR estimate of 3.5 in 2006, increasing to 3.9 in 2011, while Northern region had the highest TFR of 7.4 in 2006 declining to 6.8 in 2011. The rural/urban patterns show constant fertility at 6.9 children in rural areas for both 2006 and 2011. With regard to wealth index, the results in this paper suggest that women in the poorest wealth quintile had the highest TFR while those within the richest wealth quintiles had the least fertility outcomes. This latter finding is consistent for both 2006 and 2011. Similarly, women with a secondary and higher education had the least TFR compared to those with no education or those with a primary level of education.

## Discussion

This study sought to establish the contribution of contraception, marriage and postpartum infecundability to the total fertility rate in Uganda. Using the Aggregate Fertility Model framework [18, 23], it was possible to establish the contribution of the three parameters in explaining fertility levels in Uganda. The results in the model showed that the contribution of the three parameters namely marriage, contraception and postpartum infecundability, varies substantially given the characteristics of the woman and the two survey data sets utilized. However, the index of marriage seems to have had greater overall impact on fertility in Uganda in 2006, while the index of contraception generally had a greater effect in 2011. What is clear though, as earlier indicated, is the fact that the relative contribution of each of the three parameters varies from one population group to another. It is important that any future studies on this subject should try and establish relative significance of the different parameters in influencing the outcome variable, fertility outcome. The latter can be explored using appropriate regression techniques.

Furthermore the index of abortion was set at 1.0 in the model due to lack of data on this indicator. It is therefore important that future data collection undertakings should include questions that can allow for investigation using this fourth parameter. Nonetheless it was found in the current study that background factors including: region, residence, education, religion, and wealth status also have an influence on fertility outcomes, given that they may influence the levels of the proximate determinants.

### Study limitations

The major limitation of this study relates to the secondary nature of the data that were used. Invariably many events captured through a retrospective inquiry are often susceptible to recall bias and memory lapse. One major limitation of this study is the assumption that the index of abortion is unity and has no substantial effect on fertility estimates. This was because these data on abortion were missing and were not available for any comparable population. We observe, however that in societies that are predominantly traditional rural in nature, the assumption that induced abortion plays a minimal role would still be appropriate, and this is what guided this study. Moreover, according Bongaarts–the author of the method use [23], modeling fertility differences among populations can always be traced to variations in one or more of the proximate fertility variables. In absence of such data on abortion, therefore we acknowledge this limitation and use the rest of the components of the model to model fertility in Uganda.

## Conclusion

Therefore, inclusion of background factors in future studies has the potential and advantage of informing policy and programming on the segments of the population that require policy and programmatic intervention. The country needs to scale-up target interventions that are aimed at uplifting the education status of women and improving their economic wellbeing, because such interventions have a positive impact on fertility outcomes. In this regard, population groups with poor indices of proximate determinants of fertility would be of particular interest.

## Abbreviations

TFR: Total fertility rate
UDHS: Uganda demographic and health survey
C_m_: Index of marriage
C_c_: Index of contraception
C_a_: Index of abortion
C_i_: Index of postpartum infecundability
UBOS: Uganda bureau of statistics
ECA: Economic commission for Africa

## References

[1] Tadesse F, Headey D. Urbanization and fertility rates in Ethiopia. Washington DC: International Food Policy Research Institute (IFPRI); 2012. pp. 1-25.

[2] Erulkar A (2013). Early marriage, marital relations and intimate partner violence in Ethiopia. Int Perspect Sex Reprod Health.

[3] Asamoah BO, Agardh A, Östergren P-O. Inequality in fertility rate and modern contraceptive use among Ghanaian women from 1988–2008. Int J Equity Health. 2013, pp. 1-12. 10.1186/1475-9276-12-37 PMC3668986 23718745

[4] Gebremedhin S, Betre M (2009). Level and differentials of fertility in Awassa town, Southern Ethiopia. Afr J Reprod Health.

[5] Buyinza F, Hisali E. Microeffects of women’s education on contraceptive use and fertility: The case of uganda. J Int Dev. 2013.

[6] Burger RP, Burger R, Rossouw L (2012). The fertility transition in South Africa: A retrospective panel data analysis. Dev South Afr.

[7] Nag A, Singhal P (2013). Impact of education and age at marriage on fertility among Uttar Pradesh migrants of Ludhiana, Punjab, India. Anthropologist.

[8] Chaudhuri S (2012). The desire for sons and excess fertility: A household-level analysis of parity progression in India. Int Perspect Sex Reprod Health.

[9] Odile F, Bongaarts J (1991). Behavioural and biological determinants of fertility transition in sub-Saharan Africa. Stat Med.

[10] Darroch JE (2013). Trends in contraceptive use. Contraception.

[11] Uganda Bureau of Statistics (UBOS) and ICF International Inc. Uganda Demographic and Health Survey 2011. Kampala, Uganda: UBOS and Calverton, Maryland: ICF International Inc. 2012.

[12] Blacker J, Opiyo C, Jasseh M, Sloggett A, Ssekamatte-Ssebuliba J (2005). Fertility in Kenya and Uganda: A comparative study of trends and determinants. Popul Stud (NY).

[13] Baschieri A, Cleland J, Floyd S, Dube A, Msona A, Molesworth A (2013). Reproductive preferences and contraceptive use: A comparison of monogamous and polygamous couples in northern Malawi. J Biosoc Sci.

[14] Defo BK (2011). The importance for the MDG4 and MDG5 of addressing reproductive health issues during the second decade of life: review and analysis from times series data of 51 African countries. Afr J Reprod Health.

[15] Madhavan S, Harrison A, Sennott C (2013). Management of non-marital fertility in two South African communities. Cult Health Sex.

[16] Letamo G (1996). Contributions of the proximate determinants to fertility change in Botswana. J Biosoc Sci.

[17] Bongaarts J, Potter GR (1983). Fertility Biology And Behaviour An Analysis Of The Proximate Determinants.

[18] Bongaarts J, Odile F, Ron Lesthaeghe (1984). The Proximate Determinants of Fertility in sub-Saharan Africa. Popul Dev Rev.

[19] Bongaarts J, Stover J. The Population Council Target-Setting Model: A User’s Manual. New York: New York, Population Council 1986

[20] Bongaarts J (1982). The proximate determinants of natural marital fertility. Popul Dev Rev.

[21] Uganda Bureau of Statistics (UBOS) and Macro International Inc.: Uganda Demographic and Health Survey 2006. Calverton, Maryland, USA: UBOS and Macro International Inc. 2007.

[22] Ministry of Health Kampala, Uganda; ICF International, Calverton Maryland, USA; Centers for Disease Control and Prevention, Entebbe, Uganda; U.S. Agency for International Development, Kampala, Uganda; and WHO Uganda, Kampala, Uganda. Uganda AIDS Indicator Survey Report 2011. Ministry of Health Uganda. 2012

[23] Bongaarts J (1982). The proximate determinants of natural marital fertility. Popul Dev Rev.

[24] Inc. UB of S (UBOS) and OM. Uganda demographic and health survey 2006*.* Kampala: UBOS and Calverton. 2007

